# Multimodal fall detection for solitary individuals based on audio-video decision fusion processing

**DOI:** 10.1016/j.heliyon.2024.e29596

**Published:** 2024-04-16

**Authors:** Shiqin Jiao, Guoqi Li, Guiyang Zhang, Jiahao Zhou, Jihong Li

**Affiliations:** a School of Reliability and Systems Engineering, Beihang University, Beijing 100191, China; b Jinan Thomas School, Jinan, Shandong 250102, China

**Keywords:** Fall detection, Multimodal analysis, Audio-video fusion, Solitary individuals

## Abstract

Falls often pose significant safety risks to solitary individuals, especially the elderly. Implementing a fast and efficient fall detection system is an effective strategy to address this hidden danger. We propose a multimodal method based on audio and video. On the basis of using non-intrusive equipment, it reduces to a certain extent the false negative situation that the most commonly used video-based methods may face due to insufficient lighting conditions, exceeding the monitoring range, etc. Therefore, in the foreseeable future, methods based on audio and video fusion are expected to become the best solution for fall detection. Specifically, this article outlines the following methodology: the video-based model utilizes YOLOv7-Pose to extract key skeleton joints, which are then fed into a two stream Spatial Temporal Graph Convolutional Network (ST-GCN) for classification. Meanwhile, the audio-based model employs log-scaled mel spectrograms to capture different features, which are processed through the MobileNetV2 architecture for detection. The final decision fusion of the two results is achieved through linear weighting and Dempster-Shafer (D-S) theory. After evaluation, our multimodal fall detection method significantly outperforms the single modality method, especially the evaluation metric sensitivity increased from 81.67% in single video modality to 96.67% (linear weighting) and 97.50% (D-S theory), which emphasizing the effectiveness of integrating video and audio data to achieve more powerful and reliable fall detection in complex and diverse daily life environments.

## 1 Introduction

Contemporary economic growth often leads to various social challenges, such as an aging population, decreasing marriage rates among the youth, and higher divorce rates in married households. Consequently, there is an augmentation in the proportion of solitary family units. In China, for example, as reported in the 2021 China Statistical Yearbook, there are already over 125 million single-person households by 2020, constituting more than 25% of the total household population, as opposed to the less than 30 million in 2000, representing a mere 8.3% at that time [1]. The situation is similar in other countries. The proportion of single-person households among all Korean households increased from 15.5% in 2000 to 30.2% in 2019 [2]. In the United States, the percentage of solitary households is about 28%, and older people over the age of 65 are the main component [3].

Solitary individuals potentially encounter more safety hazards. In emergency situations, such as accidental falls, the occupants of such households may face challenges in obtaining timely assistance, which lead to serious injuries, reduced mobility, loss of independence and other irreversible consequences. As per the World Health Organization (WHO), falls rank as the second leading cause of mortality, especially among individuals aged 65 and above [4]. According to projections from the Centers for Disease Control and Prevention, there is an anticipated 30% rise in the fall-related mortality rate in the USA. The warning emphasizes that by the year 2030, there could be seven fall-related deaths per hour in the United States [5].

To mitigate the significant consequences of delayed assistance following a fall, current research are dedicated to enhancing the precision and real-time performance of fall detection at multiple levels. Commonly used fall detection technologies can be broadly classified into three categories: wearable sensor-based, environmental device-based, and camera-based systems [6].

Wearable devices include accelerometers, gyroscopes, blood glucose meters, pressure sensors, electrocardiogram (ECG), electroencephalogram (EEG), electromyogram (EOG), and more [7]. These devices, when worn, facilitate the collection of real-time physiological data from the human body, enabling the analysis of health status and real-time dynamics. Nonetheless, individuals are frequently averse to continuous sensor-wearing for the sake of comfort, especially for the elderly, who may have difficulty in mastering the operation of such devices or forgetfulness in wearing and charging them [8]. In contrast, environmental devices, such as infrared sensors, vibration sensors, Doppler radar, microphones, and others, offer an alternative approach by monitoring environmental data to detect falls. However, the installation of these devices may incur substantial unnecessary costs for individual households. Furthermore, most environmental device-based methods rely on the feedback of pressure sensors embedded in the floor, making them susceptible to false alarms as they detect the pressure changes caused by any falling object [6].

Currently, camera-based fall detection has gained popularity, owing to advancements in AI and IoT technologies. The affordability of home surveillance cameras has made them accessible to broader households, with increasing usage to monitor the well-being of the elderly, children, and pets in real-time [9]. When coupled with deep learning techniques, these cameras can significantly enhance image recognition accuracy for fall detection tasks [10]. Nevertheless, the detection process may face challenges when individuals are obscured or located within the blind area of the camera [11].

Fortunately, current advancements in home surveillance camera technology have expanded capabilities beyond unidirectional image transmission to include audio transmission and bidirectional calls. We therefore consider a multimodal approach, i.e., fusing audio classification, to compensate for the shortcomings of the visual task. Given that single-person households typically occupy relatively compact spaces, positioning the device in a specific location ensures the capture of all event sounds [12]. Audio-based fall detection leverages audio feature extracted to effectively differentiate falls from other activities.

The major contributions of this paper are outlined as follows:

(1) This paper proposes a novel fall detection framework that integrates audio and video based on non-intrusive devices, which improves the accuracy and reliability of fall detection task by taking advantage of the complementary nature of the two modalities.
(2) For different modalities, two methods are used for classification respectively: combining YOLOv7-Pose and two stream ST-GCN to achieve fall detection for videos; MobileNetV2 pre-trained model is used to achieve fall detection for audio.
(3) The multimodal decision fusion section compares the linear weighting with different weights and the D-S evidence theory, both of which prove that the multimodal fusion improves the detection effect compared to the single modality.

The subsequent sections of the paper are structured as follows: Literature review offers an extensive survey of prevailing fall detection techniques, including vision-based, audio-based, and multimodal fusion approaches. Methods introduces the methods proposed in this paper, providing detailed explanations. Experiments and results delves into the specifics of data processing and experimentation, along with a comprehensive analysis of the results. In Discussion, the strengths and limitations of the proposed method are presented, and future research directions are discussed on this basis. The conclusion and outlook for future research are described in Conclusion.

## 2 Literature review

### 2.1 Video-based methods

Video-based research stands as the predominant method for fall detection, with a primary focus on Convolutional Neural Networks (CNN), Long Short-Term Memory (LSTM), and their integrated Deep Learning (DL) models. In addition, to compensate for the lack of data and to improve computational efficiency, transfer learning (TL) is often used. Fan et al. [13] employ a fine-tuned VGG-16 network to train dynamic images of fall events. This approach facilitates the realization of both fall detection and calculation of fall duration, resulting in high-precision outcomes.

Furthermore, to enhance the accuracy of detection, human pose estimation is frequently used. A common approach involves the utilization of OpenPose for extracting 2D human pose. Subsequently, these extracted pose nodes are effectively trained using the Recurrent Neural Network (RNN) architecture, enabling the recognition of falling incidents with a cost-effective and high real-time performance [14].

To capture more features, two-stream CNNs have also been applied to fall detection. Fei et al. [15] input the optical flow into VGG-16 to capture temporal features, and input the key nodes of human skeleton into ST-GCN to capture spatial features. Subsequent steps encompass feature fusion and binary classification. Notably, this detection approach remains unaffected by variations in lighting conditions and exhibits increased robustness.

An increasing number of research is devoted to integrate both CNN and LSTM networks. Apicella and Snidaro [16] first use PoseNet for pose estimation, followed by the utilization of a pre-trained CNN to generate supplementary poses when skeletal keypoints are absent or pose estimation encounters difficulties. The ensuing classification task is carried out by LSTM. Due to the modular architecture of this approach, enhancements and adjustments can be performed more easily. Inturi et al. [5] delve into the spatial correlation of acquired skeletal keypoints through CNN, while concurrently preserving long-term dependencies through LSTM networks. It is worth noting that this study revealed the superiority of AlphaPose over OpenPose in terms of keypoint detection accuracy.

To ascertain the most effective approach for action recognition, a thorough comparative analysis is conducted [17]. The findings indicate that, for action recognition (including falls) based on skeletal data, the ST-GCN model surpasses LSTM in accuracy. Furthermore, within the framework of the ST-GCN model, the acquisition of skeletal data through YOLOv7-Pose is demonstrated to yield superior accuracy and fewer unrecognizable frames compared to YOLOv3-AlphaPose.

### 2.2 Audio-based methods

Diverse events occurring in daily life are often accompanied by distinct sounds, rendering audio classification a valuable tool for indoor activity recognition and health monitoring. Some studies initially consider conventional machine learning methods. Popescu and Mahnot [18] explore the possibility of audio-based fall detection through one-class classifiers, including nearest neighbor (OCNN), SVM (OCSVM) and Gaussian mixture (OCGM), considering the difficulty of collecting fall audio data. To better differentiate the sounds of falls from other ambient noises, all fall sounds and noise segments can be modeled using Gaussian mixture model (GMM) supervectors and classified using SVM built on a kernel between GMM supervectors, thus effectively improving the accuracy of fall detection [19].

Cheffena [20] compares diverse feature extraction methods and machine learning classifiers to identify the most effective combination for fall detection. Initially, in order to facilitate practical use, choose to use a smart phone to capture audio, and subsequent audio features are extracted through spectrogram, mel frequency cepstral coefficients (MFCCs), linear predictive coding (LPC), and matching pursuit (MP). The detection accuracy of four distinct machine learning classifiers is evaluated, including the k-nearest neighbor classifier (k-NN), SVM, least squares method (LSM), and artificial neural network (ANN). The findings indicate that the utilization of ANN classifiers in conjunction with spectrogram features results in the best detection performance.

Kim et al. [12] focus on the safety concerns of single-person households, commencing with the acquisition of audio data spanning twelve distinct categories encompassing typical daily activities and potential emergency events, which refer to situations could entail severe personal harm or fatality. Acoustic sensors are deployed for data collection. In terms of feature extraction, the adoption of log-scale mel spectrograms is predicated upon their capacity to effectively capture rich acoustic information within short-duration audio segments, with a notable emphasis on their proficiency in characterizing emergency events. Subsequently, two types of deep learning models, CNN and LSTM, are established for the classification of sound events. The outcomes underscore a significant advantage of CNN over LSTM in the classification of emergency events. It is, however, worth noting that the scope of emergency events under scrutiny in this study do not encompass falls, only similar ‘impact’.

### 2.3 Multimodal methods

In the research of fall detection, the overarching objectives have consistently revolved around enhancing detection accuracy and minimizing the incidence of false alarms. Processing and analyzing data from a single source of information often imposes constraints on the potential improvements in results. Consequently, an increasing number of studies have been exploring the integration of multiple information sources, achieved through the utilization of multimodal algorithms. This approach effectively harnesses the strengths inherent in diverse types of information, thereby optimizing the efficiency of fall detection [21].

Geertsema et al. [22] explore the utilization of video and acoustic modalities to develop a fall detection model. This model integrates motion velocity, acceleration characteristics, and peak acoustic features. Classification is performed using a SVM with a radial basis function (rbf) kernel. While the algorithm demonstrates robustness, it encounters challenges in accurately detecting low-impact falls that do not have a significant moment of impact on the floor.

In multimodal deep learning algorithms, the fusion of various modalities emerges as both central focus and significant challenge. In practical processing, the predominant methods for multimodal fusion typically align with three distinct categories: input-level, feature-level and decision-level [23], of which the latter two are more mature.

At the input-level fusion, heterogeneity poses a challenge for the fusion of different data types. Addressing this challenge, Qi et al. [24] introduce an innovative approach wherein one-dimensional time series data, sourced from wearable sensors, is encoded into a two-dimensional image format. Subsequently, this data is stacked with the two-dimensional imagery captured by cameras, facilitating input-level data fusion. This technique adeptly minimizes data information loss. Additionally, the incorporation of a federated learning (FL) training model enhances the safeguarding of user privacy.

Feature-level fusion enables the effective capture of complementary information from diverse modality data, with higher correlation between individual modalities. Amsaprabhaa et al. [25] use ST-GCN and temporal gait modeled 1D-CNN, yielding two distinct feature sets. Fall detection is achieved after using concatenative feature fusion. However, the nature of this study is still relies on a single visual data source. Therefore, it mainly focuses on processing and fusing multiple types of information rather than operating on a truly multimodal data level.

Decision-level fusion facilitates the selection of the most appropriate model for different modalities of data. This approach ensures that the modalities remain unaffected by one another, thus obviating the high requirement of early fusion for time alignment [26]. Additionally, it contributes to faster inference. The UP-Fall Detection Dataset [27] is most commonly used in multimodal fall detection research. This dataset comprises data captured through an array of sensors, including accelerometers, gyroscopes, EEG, infrared sensors, and cameras. Researchers initially apply LSTM models to analyze the sensor signals, CNN for processing video recordings, and ultimately employ a majority voting strategy for decision fusion to yield a final recognition accuracy of 96.4%.

Comprehensively comparing the advantages and disadvantages of the above methods of fall detection used based on different modalities, the results are listed in Table 1.

**Table 1:** Comparison of fall detection methods based on different modalities.

Modality	Method	Advantage	Disadvantage
Video	CNN	Spatial features can be captured efficiently	Only the static information of each frame is considered and modeling of temporal dynamics is missing
	Two-stream CNN	Spatial and temporal features can be learned separately to capture video information more comprehensively	More parameter tuning work needed
	CNN+LSTM	Suitable for tasks with long temporal dependencies	LSTM models may face the gradient vanishing problem
	ST-GCN	Especially suitable for motion recognition of skeletal joint data	Poor performance for video data with non-skeletal structure

Audio	Machine learning	Interpretable and fast training	Requires tedious manual feature engineering
	CNN	Automatic learning of features	Easily overfitted on small sample data
	LSTM	Ability to consider long temporal dependencies in audio data	More complex calculations

Multimodal	Input-level fusion	Reduced loss of data information	Data heterogeneity
	Feature-level fusion	The correlation of different modes is considered	Difficulty of time alignment
	Decision-level fusion	The most suitable model can be selected for different modality data	Incomplete integration of different modalities

## 3 Methods

**Figure 1:**
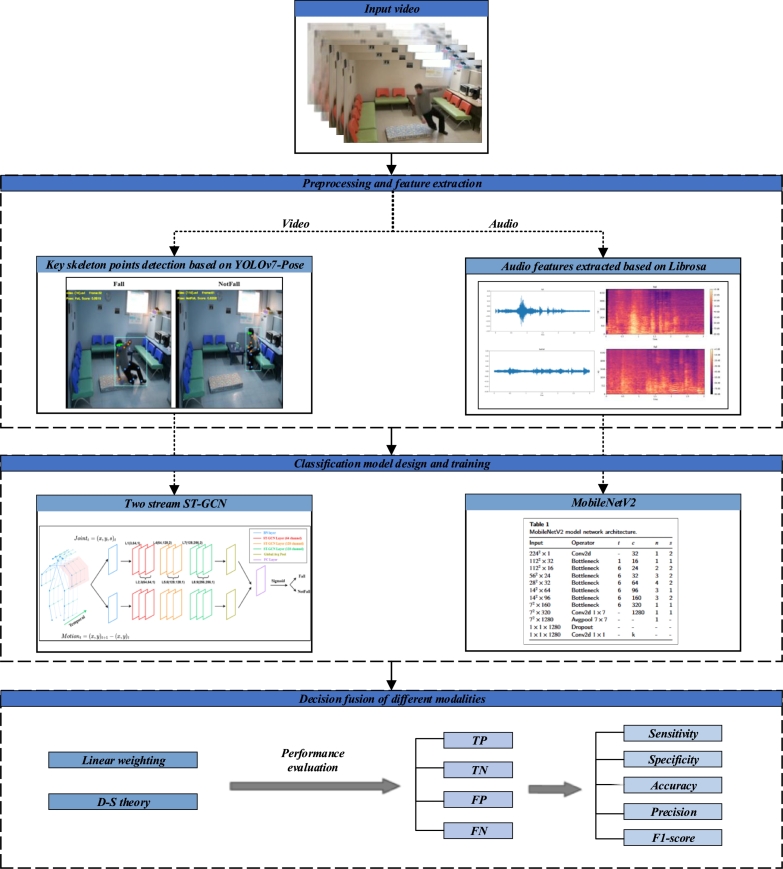
The framework of the method.

In summary, the prevailing multimodal algorithms for fall detection predominantly utilize vision-based and wearable sensor approaches. Even when acoustic data is considered, it is seldom processed using deep learning techniques. Addressing this gap, our study introduces a comprehensive multimodal fall detection algorithm that synergistically combines audio and video data within a deep learning framework. Specifically, we extract key human body skeletal nodes using the YOLOv7-Pose algorithm and analyze these data using a two stream ST-GCN. For audio, we initially obtain audio features through log-scale mel spectrograms and subsequently apply the MobileNetV2 architecture for classification. The final classification results are derived through a process of linear weighting and D-S theory for decision fusion, providing a robust solution to the problem of fall detection. The framework of proposed method is shown in Fig. 1.

### 3.1 Key skeleton points extraction based on YOLOv7-Pose

In the study of fall detection, prioritizing the detection of key skeletal nodes offers enhanced discrimination between daily activities (such as sitting down or squatting) and fall incidents, thereby improving overall accuracy. By concentrating solely on these pivotal nodes, as opposed to monitoring all body parts, computational costs are reduced, consequently leading to improved inference efficiency.

YOLO-Pose [28] represents an end-to-end human pose estimation algorithm built upon the foundation of the classical YOLOv5 object detection network model. In this architecture, CSP-darknet53 serves as the feature extraction backbone, PANet is utilized for the fusion of multiscale features. Finally, four distinct scales of detection heads are employed for predicting boxes and keypoints. This innovative approach no longer relies on encoding the original images as heatmaps but instead associates all keypoints of an individual with anchor points. This modification improves the algorithm's ability to respond effectively to occlusion scenarios.

**Figure 2:**
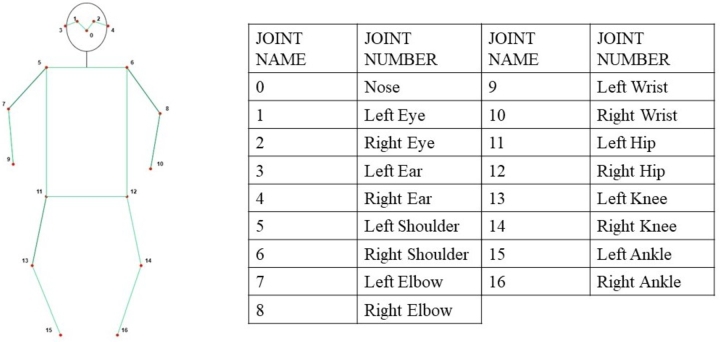
Skeleton joint points.

For each anchor, the key point head recognizes 17 key skeletal points for each individual (see Fig. 2), with each node *v* comprising both position and confidence information, denoted as v=(x,y,score), totaling 51 elements. In addition the box header predicts six elements: the center position (Cx,Cy), the width *W*, the height *H*, the confidence of the bounding box, and the confidence of the class. Finally, the overall prediction vector $P_{v}$ is defined in Eq. (1).

(1) $$P_{v}=\left\{C_{x},C_{y},W,H,box_{conf},class_{conf},K_{x}^{1},...,K_{x}^{17},K_{y}^{17},K_{conf}^{17}\right\}$$

The most recent iteration, YOLOv7-Pose, has transitioned to the YOLOv7 network architecture [29], incorporating the Efficient Layer Aggregation Networks (ELAN) and Extended-ELAN (E-ELAN) architectures in the backbone segment. This move replaces the previously utilized CSP-darknet53 of YOLOv5, resulting in improved efficiency.

**Figure 3:**
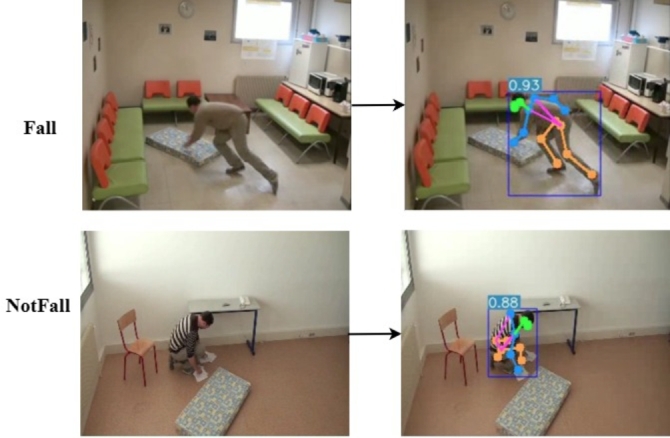
Extracting skeleton nodes illustration based on YOLOv7-Pose, the dataset from Charfi et al. [30].

Fig. 3 illustrates the output of key skeletal point extraction using YOLOv7-Pose. The figure demonstrates the algorithm's robustness, highlighting its effectiveness in recognizing key nodes even when they are occluded.

### 3.2 Fall detection based on ST-GCN

ST-GCN [31] consists of a stack of multilayer spatial and temporal convolutional layers, enabling the network to acquire a deeper understanding of complex spatial and temporal dependencies. When processing skeleton action data, this architecture can efficiently captures and comprehends the spatial structure and temporal dynamics within human skeletons, and analyzes spatial and temporal relationships within graph-structured data.

Specifically, ST-GCN utilizes GCN for the task of human action recognition based on skeletal sequences, where the human skeleton sequence is defined as a spatio-temporal graph G=(V,E). The node set $V=\left\{v_{t,i}|t=1,2,...,T,\;i=1,2,...,17\right\}$ contains all the information of the sequence of skeleton points, $v_{t,i}$ representing the *i*-th joint on frame *t*; while the edge *E* includes the connection information of all the skeleton points in the same frame $E_{s}=\left\{v_{ti}v_{tj}|(i,j)\in H\right\}$, and the one of the same skeleton points between the two neighboring frames $E_{F}=\left\{v_{ti}v_{(t+1)j}\right\}$, where *H* is a set of naturally connected human joints.

For the spatial information of skeleton points in a single frame, the traditional convolutional network can be extended to spatial graph convolution, which is expressed as Eq. (2).

(2) $$f_{out}(v_{ti})=\sum_{v_{tj}\in B(v_{ti})}\frac{1}{Z_{ti}(v_{tj})}f_{in}(p(v_{ti},v_{tj}))\cdot w(v_{ti},v_{tj})$$

The normalization term $Z_{ti}(v_{tj})$ is used to balance the contribution of different subsets to the output. In neighbor set $B(v_{ti})=\left\{v_{tj}|d(v_{tj},v_{ti})\leq 1\right\}$, $d(v_{tj},v_{ti})$ represents the minimum length of any path from $v_{tj}$ to $v_{ti}$, which means that the sampling function $p=(v_{ti},v_{tj})$ samples only directly adjacent nodes. And the weight function $w=(v_{ti},v_{tj})$ is achieved by dividing the nodes by different partition strategies.

Afterwards, the spatial graph CNN is further extended to the spatio-temporal domain, wherein concept of adjacency is extended to temporally connected nodes, which can be expressed as Eq. (3).

(3) $$B(v_{ti})=\left\{v_{qj}|d(v_{tj},v_{ti})\leq K,\left|q-t\right|\leq \left\lfloor \Gamma /2\right\rfloor \right\}$$

**Figure 4:**
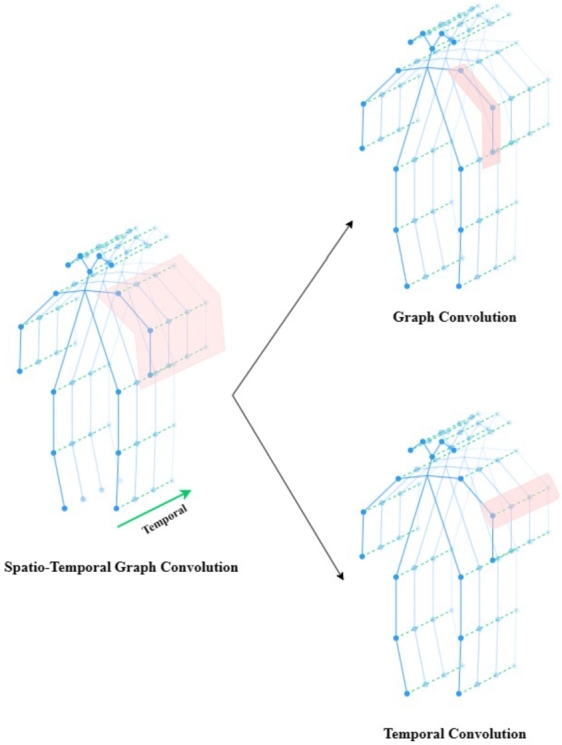
Spatio-temporal graph convolution.

In this formula, Γ is the temporal kernel size, thus defining neighbor nodes at the temporal level as before and after in frame distance less than Γ/2. Fig. 4 shows the spatio-temporal graph convolution representation on the skeleton sequence.

**Figure 5:**
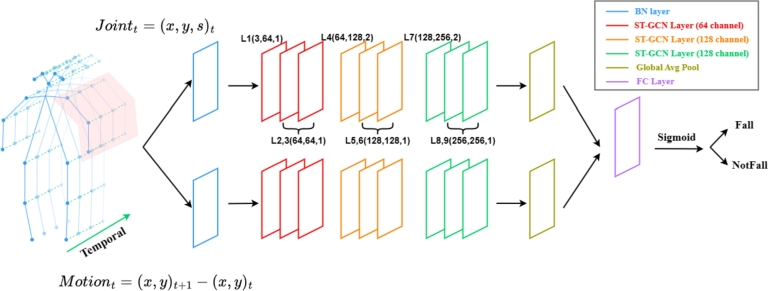
Two stream ST-GCN network. For each stream, there are a total of nine ST-GCN layers (L1-L9). The three numbers of each layer represent the number of input channels, the number of output channels and the stride, respectively.

**Figure 6:**
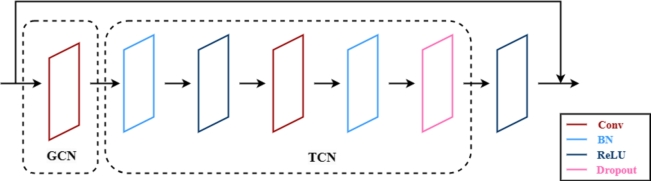
ST-GCN layer.

For network modeling, two stream ST-GCN structure is used to better capture motion information, shown in Fig. 5. While retaining the original node information input stream, a supplementary motion stream is added, that represents the change in node coordinates between consecutive frames. Both streams are concurrently processed within the same ST-GCN framework, comprised of nine ST-GCN layers. After spatio-temporal convolution of all ST-GCN layers, the feature dimension (spatial dimension) of the joints will increase and the keyframe dimension (temporal dimension) will decrease. This is due to the fact that for the action of falling only a few phases are needed to describe it, but the variation of the action within each phase is complex. To avoid overfitting, we implement a dropout regularization strategy set to 0.5, and the strides of the 4-th and the 7-th temporal convolution layers are set to 2 as pooling layer. The Graph Convolutional Network (GCN) and Temporal Convolutional Network (TCN) modules will be traversed within the ST-GCN layer, shown in Fig. 6. Finally, the outputs from the two streams are integrated at a fully connected layer.

Cross entropy loss is widely used in classification tasks. Consequently, the binary cross entropy (BCE) is adopted as the loss function for the validation of this binary classification model, and the specific calculation process is shown in Eq. (4).

(4) $$Loss=-\frac{1}{N}\sum_{n=1}^{N}(y^{\left(n\right)}log({\overset{ˆ}{y}}^{\left(n\right)})+(1-y^{\left(n\right)})log(1-{\overset{ˆ}{y}}^{\left(n\right)})$$

Where *N* denotes the batch size. For the *n*-th sample, $y^{\left(n\right)}$ represents the true label and takes the value 0 or 1 (fall or notfall), while ${\overset{ˆ}{y}}^{\left(n\right)}$ represents the predicted outcome, usually a probability between 0 and 1.

### 3.3 Audio classification based on CNN

**Figure 7:**
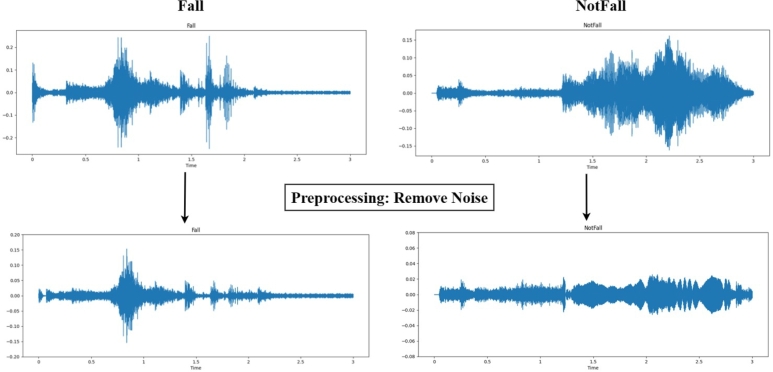
Comparison of sound waveforms before and after preprocessing.

The acoustic feature associated with an individual's fall typically exhibits distinct characteristics when compared to sounds from daily activities. This suggests that audio classification offers a viable approach for fall detection. Although a diverse audio dataset can enhance model robustness, excessive noise may affect the model's classification efficacy. Therefore, the audio extracted from the video needs to be preprocessed first, including the removal of irrelevant noise segments and human voices. Fig. 7 shows the change of sound waveforms after preprocessing. The comparison shows that preprocessing effectively accentuates the acoustic variations during falls and eliminates segments of daily activities that could potentially compromise the classification outcomes.

**Figure 8:**
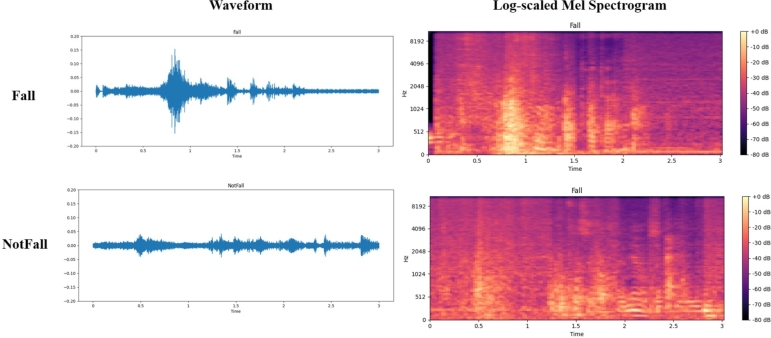
Waveforms and log-scaled mel spectrograms.

In terms of audio feature extraction, log-scaled mel spectrograms (shown in Fig. 8) use human auditory features to interpret audio signals, including time domain and spectral domain features. This representation is similar to the extracted image features, making it easy to subsequently combine it with network architectures commonly used for image recognition tasks, such as CNNs.

According to the pre-trained audio neural networks compared by Kong et al. [32], it can be found that among the architectures analyzed, ResNets demonstrates superior accuracy, while MobileNets shows noteworthy efficiency in audio classification task. Given the limited volume of audio data utilized in this research and the simplicity of the task, we choose to use the classical lightweight network MobileNetV2 [33] to mitigate potential overfitting and to ensure computational efficiency. The detailed network structure is shown in Table 2.

**Table 2:** MobileNetV2 model network architecture.

Input	Operator	*t*	*c*	*n*	*s*
224^2^ × 1	Conv2d	-	32	1	2
112^2^ × 32	Bottleneck	1	16	1	1
112^2^ × 16	Bottleneck	6	24	2	2
56^2^ × 24	Bottleneck	6	32	3	2
28^2^ × 32	Bottleneck	6	64	4	2
14^2^ × 64	Bottleneck	6	96	3	1
14^2^ × 96	Bottleneck	6	160	3	2
7^2^ × 160	Bottleneck	6	320	1	1
7^2^ × 320	Conv2d 1 × 7	-	1280	1	1
7^2^ × 1280	Avgpool 7 × 7	-	-	1	-
1 × 1 × 1280	Dropout	-	-	-	-
1 × 1 × 1280	Conv2d 1 × 1	-	k	-	-

MobileNet uses depth-separable convolution instead of the traditional standard convolution operation. It consists of two steps, deep convolution and point-by-point convolution, which helps to reduce the number of parameters and computational cost [34]. MobileNetV2 is a further optimized version of the series, which improves the nonlinear modeling capability by introducing inverted residual structure and linear activation function, and further reduces the fully connected layers through global average pooling, making the model more lightweight and efficient for embedded devices.

For model evaluation, we use *k*-fold cross-validation owing to the constraints of the dataset size. This method mitigates uncertainties associated with data partitioning and curbs the risk of overfitting. In our study, *k* is set at 5.

### 3.4 Multimodal decision fusion

In decision fusion, linear weighted summation is the predominant method. Upon independently training the video and audio networks, we derive two distinct sets of predictions $R_{video}(a_{1},a_{2},...,a_{m})$ and $R_{audio}(b_{1},b_{2},...,b_{m})$, where *a* and *b* represent the probabilistic predicted values for the *m*-th video. The predicted probabilities are linearly weighted, calculated as Eq. (5).

(5) $$c_{m}=\alpha \cdot a_{m}+(1-\alpha )\cdot b_{m}$$

Finally, we get the decision fusion result $R_{fusion}(c_{1},c_{2},...,c_{m})$, where *α* denotes the weight of the video modality. Since visual-based fall detection has a wider range of application scenarios, and the objective of this study is to supplement video detection with audio, we set 0.6≤α≤0.9.

Furthermore, to augment the efficacy of information fusion, the incorporation of the D-S evidence theory is contemplated. This framework offers enhanced flexibility, fusing different information via the Dempster combination rule, which no longer relies on a priori probability [35].

The theory states that Θ constitutes a recognition framework, including all independent and mutually exclusive events, seen in Eq. (6).

(6) $$\Theta =\left\{\theta_{1},\theta_{2}\right\}$$

In this study it is specified that $\theta_{1}$ is a fall and $\theta_{2}$ is a non-fall.

$2^{\theta }$ denotes the set of all possible hypotheses, as shown in Eq. (7).

(7) $$2^{\theta }=\left\{\emptyset ,\left\{\theta_{1}\right\},\left\{\theta_{2}\right\},\left\{\theta_{1},\theta_{2}\right\}\right\}$$

Assigning a probability for each hypothesis is called basic probability assignment (BPA), and the BPA function, also called the mass function, specifies that the sum of the mass function values for all hypotheses is one, seen in Eq. (8).

(8) $$\sum_{A\subseteq 2^{\Theta }}m(A)=1$$

For each hypothesis, the corresponding mass function produces a belief function bel(A) and a plausibility function pl(A). Eq. (9) and Eq. (10) define bel(A) and pl(A), respectively.

(9) $$bel(A)=\sum_{B|B\subseteq A}m(B)$$

(10) $$pl(A)=\sum_{B|B\cap A\neq \;\emptyset }m(B)$$

The belief function bel(A) for a hypothesis *A* is defined as the sum of the mass values for all hypotheses *B* that are truly subsets of *A*.

**Figure 9:**
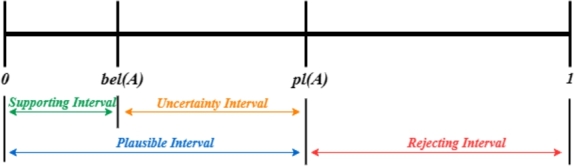
The relation diagram of *bel*(*A*) and *pl*(*A*).

The plausibility function pl(A) for hypothesis *A* is quantified as the aggregate of the mass function values for all hypotheses *B* that intersect with *A* and are non-empty. The interval [bel(A),pl(A)], delineating the range of hypothesis *A* constitutes its credibility interval, as illustrated in Fig. 9.

In this paper, we assume that the mass functions of the video and audio modalities are $m_{1}$ and $m_{2}$, respectively, then the probability for hypothesis *A* is calculated as Eq. (11).

(11) $$m_{1,2}(A)=(m_{1}⊕m_{2})(A)=\frac{1}{1-K}\sum_{B\cap C=A\neq \emptyset }\left[m_{1}(B)\cdot m_{2}(C)\right]$$

Where *K* is the conflict measure, calculated as Eq. (12).

(12) $$K=\sum_{B\cap C\neq \emptyset }m_{1}(B)\cdot m_{2}(C)=1-\sum_{B\cap C\neq \emptyset }m_{1}(B)\cdot m_{2}(C)$$

### 3.5 Performance analysis

Before evaluating the model's performance, it is essential to first define the following parameters [36]:

1. True positives (TP): Fall and is correctly detected.
2. True negatives (TN): Not fall and correctly detected.
3. False positives (FP): Not fall but detected as fall.
4. False negatives (FN): Fall but detected as not fall.

To evaluate the performance of the implemented models, five categorical performance metrics are typically used [37]:

1. Sensitivity, reflecting the capability to identify fall events.
2. Specificity, gauging the capacity to discern non-fall events.
3. Accuracy, representing the overall rate of correct detections.
4. Precision, evaluating the reliability of the detected falls.
5. F1-score, offering a comprehensive assessment measure that takes into account both misses and false positives.

The calculation process is as follows, where *ω* represents the number of events in which such a situation occurs:

$$Sensitivity=\frac{\omega_{TP}}{\omega_{TP}+\omega_{FN}}$$

$$Specificity=\frac{\omega_{TN}}{\omega_{TN}+\omega_{FP}}$$

$$Accuracy=\frac{\omega_{TP}+\omega_{TN}}{\omega_{TP}+\omega_{TN}+\omega_{FP}+\omega_{FN}}$$

$$Precisino=\frac{\omega_{TP}}{\omega_{TP}+\omega_{FP}}$$

$$F1-score=\frac{2\omega_{TP}}{2\omega_{TP}+\omega_{FP}+\omega_{FN}}$$

## 4 Experiments and results

### 4.1 Dataset and preprocess

We select the widely-recognized Le2i Fall Datasets [30], which contains a total of 190 videos, with 130 showcasing falls. Each video captures the activities of a single individual with a resolution of 320*240 and a frame rate of 25 fps. The dataset contains variable illumination as well as shadows and reflections that can be detected as moving objects, and typical difficulties such as occlusions and cluttered backgrounds. The tested persons wearing clothes of different colors and textures have performed daily activities (walking in different directions, sitting down, standing up, squatting, doing housework, moving chairs, etc.) and falls (falling while walking, falling while sitting, falling off steps, etc.). All activities are performed in different directions without taking into account the camera perspective.

Since the brief duration of falls in real-life scenarios, the number of fall frames contained in the videos in the dataset is much less than that of daily activities. In order to avoid the datasets being too unbalanced to affect the training results, the datasets are divided twice to ensure that all the videos have a duration of 3 s. Among them, there are 130 video segments for both falls and non-falls; and the number of frames for the falls is 9639 and the one for the non-falls is 9659. Such a relatively balanced sample size can improve the sensitivity of the model and thus better detect falls. Subsequently, the data is split into training and testing sets at an 8:2 ratio.

For audio, the sounds of the re-segmented datasets are extracted by FFmpeg as the original audio datasets. We first used the open source software Audacity to remove irrelevant vocal noise (such as the laughter of onlookers) in the clips to avoid affecting the training results. Due to the limited quantity of audio data, it is easy to overfitting in training. To counteract this situation, data enhancement is performed. We use five enhancement method: adding white noise, time domain lengthening, time domain shortening, increasing volume and decreasing volume. By performing different audio transformations, the model is more likely to learn a more robust audio data representation under different environmental conditions, thereby enhancing the model's adaptability. Finally a total of 260×6=1560 augmented audio datasets are generated.

### 4.2 Experiments

**Figure 10:**
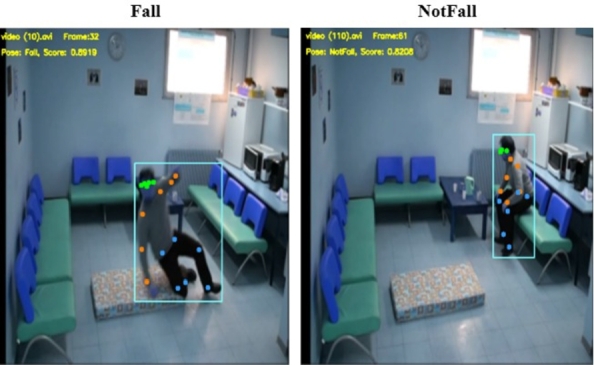
Extracting key skeletal joint points illustration based on YOLOv7-Pose.

**Figure 11:**
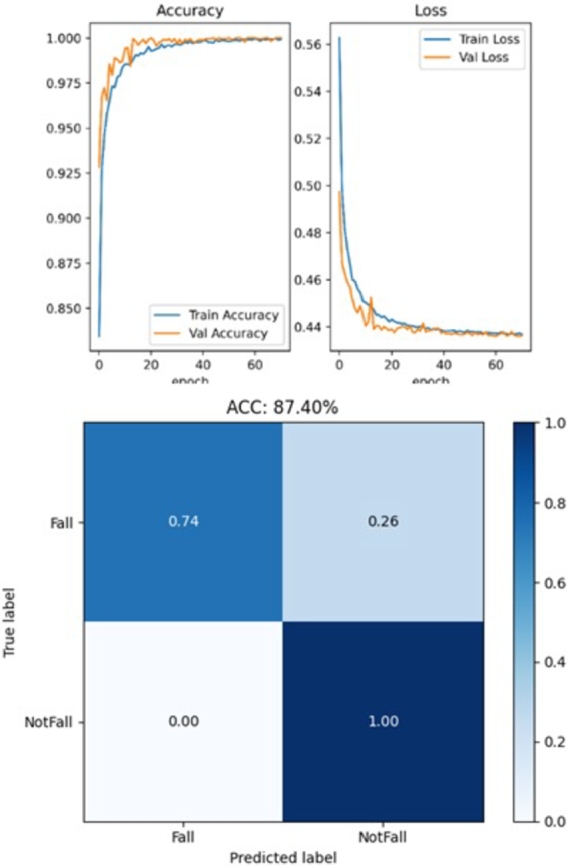
Video-based model training results.

For video, the coordinates of 17 key nodes are initially extracted from each frame using the YOLOv7-Pose algorithm, shown in Fig. 10. Subsequent data processing involves eliminating blank frames and normalizing the joint coordinates. The two stream ST-GCN network uses the Adam optimizer during training, and the learning rate is set to 0.001. The model tends to converge after about 60 epochs, and evaluation of the model performance are given in the confusion matrix seen in Fig. 11.

For audio, the audio features are extracted by log-scaled mel spectrogram. The model is trained with the help of two different CNN pre-trained models: MobileNetV2 (learning rate = 0.001 and ResNet18 (learning rate = 0.0001). In order to prevent overfitting, the L2 regularization term is set for the Adam optimizer. Subsequent *k*-fold cross-validation is used and the performance evaluation metrics of the model are shown in Table 3.

**Table 3:** Comparison of MobileNetV2 and ResNet18 model performance.

Model	Fold	Sensitivity	Specificity	Accuracy	Precision	F1-score
**ResNet18**	1	98.98%	98.20%	98.59%	98.22%	98.60%
	2	99.92%	99.84%	99.88%	99.84%	99.88%
	3	100.00%	100.00%	100.00%	100.00%	100.00%
	4	100.00%	100.00%	100.00%	100.00%	100.00%
	5	100.00%	100.00%	100.00%	100.00%	100.00%
**Average**		99.78%	99.61%	99.69%	99.61%	99.70%

**MobileNetV2**	1	97.34%	96.88%	97.11%	96.89%	97.12%
	2	99.61%	98.67%	99.14%	98.68%	99.14%
	3	99.77%	99.61%	99.69%	99.69%	99.69%
	4	99.92%	99.69%	99.80%	99.69%	99.80%
	5	100.00%	100.00%	100.00%	100.00%	100.00%
**Average**		99.33%	98.97%	99.15%	98.97%	99.15%

The comparison results analysis shows that, while the ResNet18 exhibits a marginally superior model performance relative to that of MobileNetV2, the discrepancy is minimal. Notably, MobileNetV2 demonstrates pronounced benefits with respect to its compact model footprint and computational expediency. These advantages highlight its potential utility in applications where model efficiency is critical, without significantly compromising prediction accuracy.

### 4.3 Inference results

To test the generalization capability of the model, a batch of data designated for inferential validation is regenerated from the original datasets. This subset includes 240 video recordings. The ensuing procedural methodology for data processing is shown in Table 4.

**Table 4:** New inferential data generation grouping process.

Data name	Label	Video-based process	Audio-based process
Video(1)-(20)	Fall	Light brightness down (factor=0.5)	Add noise
Video(21)-(40)			Change time domain
Video(41)-(60)			Change volume
Video(61)-(80)		Image flip + Light brightness down (factor=0.8)	Add noise
Video(81)-(100)			Change time domain
Video(101)-(120)			Change volume

Video(121)-(140)	NotFall	Light brightness down (factor=0.5)	Add noise
Video(141)-(160)			Change time domain
Video(161)-(180)			Change volume
Video(181)-(200)		Image flip + Light brightness down (factor=0.8)	Add noise
Video(201)-(220)			Change time domain
Video(221)-(240)			Change volume

By deploying the previously optimized network, which has undergone rigorous training, we process the data to obtain predictive results. The inferential outcomes for the video frames are systematically obtained in Table 5.

**Table 5:** Inference results for video frame-level.

Parameters		Evaluation metric	
TP	3539	Sensitivity	68.71%
FP	6	Specificity	99.89%
TN	5287	Accuracy	84.51%
FN	1612	Precision	99.83%
Total	10444	F1-score	81.39%

Since the judgment is now based on video frames, it is necessary to transmute these frame-level detections into an integrated video-level detections. This synthesis is critical for the fusion of inferential outcomes derived from audiovisual data. Recognizing that a fall constitutes a temporally contiguous event, we have instituted a criterion whereby a video is classified as depicting a fall if a sequence of 15 consecutive frames is identified as ‘Fall’. The confidence metric associated with this event classification is derived from the maximal value amongst the mean confidence scores of the said contiguous frames. The findings that emanate from this refined evaluative approach are documented in Table 6.

**Table 6:** Inference results for video-level.

Parameters		Evaluation metric	
TP	98	Sensitivity	81.67%
FP	0	Specificity	100.00%
TN	120	Accuracy	90.83%
FN	22	Precision	100.00%
Total	240	F1-score	89.91%

The inference outputs obtained using the audio-based network previously fine-tuned to peak performance are shown in Table 7.

**Table 7:** Inference results for audio.

Parameters		Evaluation metric	
TP	114	Sensitivity	95.00%
FP	2	Specificity	98.33%
TN	118	Accuracy	96.67%
FN	6	Precision	98.28%
Total	240	F1-score	96.61%

**Figure 12:**
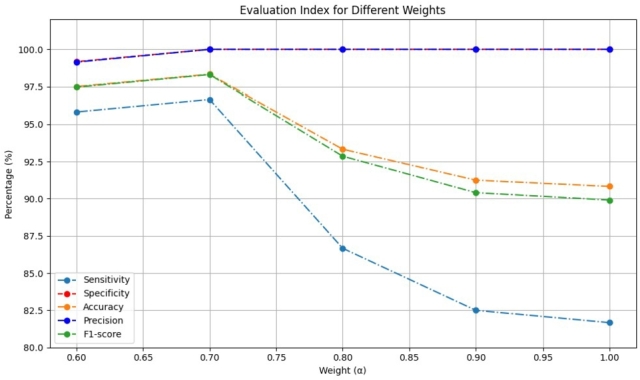
Impact of different weights on results.

A linear weighted fusion strategy is employed to synthesize the inference results from both video and audio modalities. The weighting attributed to the video modality is variably adjusted from 0.6 to 0.9, and the combined inference results are shown in Fig. 12.

### 4.4 Results analysis

In the video modality, by comparing Table 5 and Table 6, it can be found that compared with fall detection based on video frame-level, the sensitivity and accuracy of video-level detection are significantly improved. This is because there may be some action frames that are not completely fallen during the analysis process and are difficult to identify. In reality, falling is a continuous process, so video-level detection is more reasonable and efficient.

However, it is comprehensively found that the sensitivity of model based on video detection always performs unsatisfactorily and has a high false negative rate, which are usually due to the challenges in the comprehensive recognition of the human skeletal structure. For example, scenarios in which the majority of the human form is obscured by furnishings or a fall occurring at the periphery of the camera's field of view will impede the model's capacity to recognize critical skeleton nodes, thereby precluding accurate fall detection. Furthermore, lighting conditions have a significant impact on prediction performance. In dim illumination, coupled with subjects attired in darker hues, the differentiation between the individual and the backdrop becomes difficult, thus adversely affecting the recognition process. But it is gratifying that the specificity and precision of the model perform ideally, proving that video-based fall detection can effectively identify non-fall situations, and the identified fall events are very reliable with almost no false positives.

In the audio modality, by analyzing the four types of metrics in Table 7, it can be found that the overall performance of the audio-based fall detection model is relatively good, but there are still very few false positives and false negatives. This may be because when a person falls slowly or from a relatively low elevation, such as sitting on a chair, the resultant sound may be faint, posing difficulty in accurate fall recognition. In addition, the current model's capacity to differentiate between the acoustics of a fall and other analogous sounds remain limited. Sounds that mimic the impact of a fall, such as abrupt stomping or stumbling, can be erroneously interpreted as a fall by the system, leading to misjudgment.

In multimodality, by observing Fig. 12, it can be seen that when α=0.7, the effectiveness of the detection model reaches its peak. At this time, the audio input effectively improves the false negatives that occur in video-based detection. However, allocating an excessive weight to the audio modality (e.g., 0.4), contrarily engendered a negative impact, wherein the wrong results of the audio inference adversely affect the fusion detection.

In addition, we also consider using D-S theory for decision fusion. Table 8 compares our method with existing methods on the Le2i dataset. It can be seen that our multimodal method shows certain advantages in various performances.

**Table 8:** Experimental comparison results on the Le2i dataset.

	Method	Sensitivity	Specificity	Accuracy	Precision	F1-score
Poonsri and Chiracharit [38]	GMM	93.18%	64.29%	86.21%	89.13%	91.11%
Wang et al. [39]	YOLO-OpenPose+MLP+RF	96.51%	97.37%	96.91%	97.65%	97.08%
Youssfi Alaoui et al. [40]	V2V-PoseNet+SVM	**100.0%**	87.33%	92.30%	83.60%	91.06%
Zheng and Liu [41]	AlphaPose+ST-GCN	96.71%	96.81%	96.86%	97.01%	96.77%
Our methods	Linear weighting(*α* = 0.7)	96.67%	**100.0**%	**98.33%**	**100.0%**	98.31%
	D-S theory	97.50%	99.17%	**98.33%**	99.15%	**98.32%**

The two decision fusion methods also have their own characteristics: linear weighted fusion can select the parameter setting with the best performance, and will be more affected by high-weight information source in the case of information conflicts. It is found in the table that this method well reflects the zero false positives of video modality, while the metric sensitivity is significantly improved. The various metrics of D-S decision theory have also been improved and the performance is relatively more balanced. Among them, sensitivity performances better because this method can better weigh the contributions of different information sources, thereby enabling effective merging in the case of conflicts between information sources.

## 5 Discussion

The multimodal fall detection method based on audio and video proposed in this study only relies on non-invasive devices. Compared with wearable devices and environmental sensors, it will have almost no impact on the normal life of the person being detected. In addition, this method comprehensively considers the detection results of different information sources, which can effectively improve the performance of the results and reduce the false negatives and false positives compared with a single modality.

However, it needs to be acknowledged that our approach also has certain limitations. Compared with the strong generalization ability of video-based methods, audio-based methods may cause poor detection performance due to the influence of the environment, such as different floor materials or background noise, which will greatly reduce the detection effect. Although our approach is originally designed to use audio as an auxiliary to video, it is still uncertain how well this multimodal model will perform in other scenarios. In addition, our method is currently not deployed in actual scene devices for verification, and it is not possible to delve into practical issues such as inference speed and data transmission.

In the future, we will further supplement and improve the existing research. First, we need to form an end-to-end detection system with this method, which can be deployed and verified in practice; and we will consider cooperating with nursing homes, hospitals and other enterprises to obtain more real data; in addition, methods will be optimized, including more thorough feature-level fusion and anonymization that considers privacy security to facilitate model promotion.

## 6 Conclusion

Falls are a major safety concern in single-person households, and older adults are particularly vulnerable. Consequently, the development of precise and efficient fall detection methodologies has emerged as a focal point of contemporary research. Furthermore, the integration of multimodal technologies is anticipated to substantially enhance detection accuracy and robustness by leveraging the complementary strengths of diverse modalities.

In this study, we explore ways to combine video and audio modalities based on non-invasive devices to enhance fall detection. Specifically, we employ the YOLOv7-Pose algorithm to identify critical skeletal nodes in video data, while two stream ST-GCN extracts pertinent spatial and temporal information to render a frame-level decision. This decision is then aggregated to formulate a video-level conclusion. Concurrently, in the audio domain, we implement a data augmentation technique to enrich our dataset, followed by the extraction of features using the log-scaled mel spectrogram. Subsequent analysis employs *k*-fold cross-validation to contrast the performance metrics of the MobileNetV2 and ResNet18 models. Despite the similar accuracy of the two architectures, MobileNetV2 is ultimately selected for its superior computational efficiency and more compact model size.

In order to test the generalization ability of the model, the original dataset is subjected to a variety of transformations including dimming the brightness, mirror inversion, changing the time domain, changing the volume and adding noise. The subsequent analysis reveals that the video modality struggles to accurately detect falls under conditions of low illumination or significant obstruction of the human figure. The efficacy of the inference results is significantly enhanced through the application of decision-level fusion techniques combining audio modalities, with optimal outcomes observed when the video modality is assigned a weight of 0.7. Furthermore, it is determined that decision fusion employing D-S theory yields a more balanced performance in the model compared to linear weighting methods.

We believe that multimodal audio-visual fusion methods, due to their advantages, are expected to become the optimal means of fall detection. In future research, we expect to apply our work to implement automatic fall detection systems in actual surveillance camera networks; and further optimize and improve the model based on actual data, and consider integrating more thorough feature-level fusion technology, paying special attention to time alignment methods; additionally there will be an emphasis on addressing privacy and data security issues, including the incorporation of image and audio anonymization techniques.

## CRediT authorship contribution statement

**Shiqin Jiao:** Writing – original draft, Methodology, Formal analysis, Conceptualization. **Guoqi Li:** Writing – review & editing, Supervision, Methodology, Conceptualization. **Guiyang Zhang:** Visualization, Validation, Formal analysis. **Jiahao Zhou:** Writing – original draft, Investigation. **Jihong Li:** Writing – review & editing, Supervision.

## Declaration of Competing Interest

The authors declare that they have no known competing financial interests or personal relationships that could have appeared to influence the work reported in this paper.

## Data Availability

Data will be made available on request.

## References

[1] Long H., Shi S., Tang Z., Zhang S. (2022). Does living alone increase the consumption of social resources?. Environ. Sci. Pollut. Res..

[2] Jung H., Kim J.H., Hong G. (2023). Impacts of the Covid-19 crisis on single-person households in South Korea. J. Asian Econ..

[3] Chamie J. (2023). Population Levels, Trends, and Differentials: More Important Population Matters.

[4] Mozaffari N., Rezazadeh J., Farahbakhsh R., Yazdani S., Sandrasegaran K. (2019). Practical fall detection based on iot technologies: a survey. Int. Things.

[5] Inturi A.R., Manikandan V., Garrapally V. (2023). A novel vision-based fall detection scheme using keypoints of human skeleton with long short-term memory network. Arab. J. Sci. Eng..

[6] Mubashir M., Shao L., Seed L. (2013). A survey on fall detection: principles and approaches. Neurocomputing.

[7] Wang X., Ellul J., Azzopardi G. (2020). Elderly fall detection systems: a literature survey. Front. Robot. AI.

[8] Droghini D., Squartini S., Principi E., Gabrielli L., Piazza F. (2019). Audio metric learning by using Siamese autoencoders for one-shot human fall detection. IEEE Trans. Emerg. Top. Comput. Intell..

[9] De Miguel K., Brunete A., Hernando M., Gambao E. (2017). Home camera-based fall detection system for the elderly. Sensors.

[10] Alam E., Sufian A., Dutta P., Leo M. (2022). Vision-based human fall detection systems using deep learning: a review. Comput. Biol. Med..

[11] Li Y., Ho K., Popescu M. (2012). A microphone array system for automatic fall detection. IEEE Trans. Biomed. Eng..

[12] Kim J., Min K., Jung M., Chi S. (2020). Occupant behavior monitoring and emergency event detection in single-person households using deep learning-based sound recognition. Build. Environ..

[13] Fan Y., Levine M.D., Wen G., Qiu S. (2017). A deep neural network for real-time detection of falling humans in naturally occurring scenes. Neurocomputing.

[14] Hasan M.M., Islam M.S., Abdullah S. (2019). 2019 IEEE International Conference on Robotics, Automation, Artificial-Intelligence and Internet-of-Things (RAAICON).

[15] Fei K., Wang C., Zhang J., Liu Y., Xie X., Tu Z. (2023). Flow-pose net: an effective two-stream network for fall detection. Vis. Comput..

[16] Apicella A., Snidaro L. (2021). Pattern Recognition. ICPR International Workshops and Challenges: Virtual Event, Proceedings, Part II.

[17] Dao N., Le T., Tran H., Nguyen Y., Duy T. (2022). The combination of face identification and action recognition for fall detection. J. Sci. Technol. Iss. Inf. Commun. Technol..

[18] Popescu M., Mahnot A. (2009). 2009 Annual International Conference of the Ieee Engineering in Medicine and Biology Society.

[19] Zhuang X., Huang J., Potamianos G., Hasegawa-Johnson M. (2009). 2009 IEEE International Conference on Acoustics, Speech and Signal Processing.

[20] Cheffena M. (2015). Fall detection using smartphone audio features. IEEE J. Biomed. Health Inform..

[21] Xefteris V.-R., Tsanousa A., Meditskos G., Vrochidis S., Kompatsiaris I. (2021). Performance, challenges, and limitations in multimodal fall detection systems: a review. IEEE Sens. J..

[22] Geertsema E.E., Visser G.H., Viergever M.A., Kalitzin S.N. (2019). Automated remote fall detection using impact features from video and audio. J. Biomech..

[23] Poria S., Cambria E., Bajpai R., Hussain A. (2017). A review of affective computing: from unimodal analysis to multimodal fusion. Inf. Fusion.

[24] Qi P., Chiaro D., Piccialli F. (2023). Fl-fd: federated learning-based fall detection with multimodal data fusion. Inf. Fusion.

[25] Amsaprabhaa M. (2023). Multimodal spatiotemporal skeletal kinematic gait feature fusion for vision-based fall detection. Expert Syst. Appl..

[26] Wang X. (2023). Egofalls: a visual-audio dataset and benchmark for fall detection using egocentric cameras. 10.48550/arXiv.2309.04579.

[27] Martínez-Villaseñor L., Ponce H., Perez-Daniel K. (2019). 2019 IEEE International Conference on Systems, Man and Cybernetics (SMC).

[28] Maji D., Nagori S., Mathew M., Poddar Yolo-pose D. (2022). Proceedings of the IEEE/CVF Conference on Computer Vision and Pattern Recognition.

[29] Wang C.-Y., Bochkovskiy A., Liao H.-Y.M. (2023). Proceedings of the IEEE/CVF Conference on Computer Vision and Pattern Recognition.

[30] Charfi I., Miteran J., Dubois J., Atri M., Tourki R. (2012). 2012 Eighth International Conference on Signal Image Technology and Internet Based Systems.

[31] Yan S., Xiong Y., Lin D. (2018). Proceedings of the AAAI Conference on Artificial Intelligence.

[32] Kong Q., Cao Y., Iqbal T., Wang Y., Wang W., Plumbley M.D. (2020). Panns: large-scale pretrained audio neural networks for audio pattern recognition. IEEE/ACM Trans. Audio Speech Lang. Process..

[33] Sandler M., Howard A., Zhu M., Zhmoginov A., Chen L.-C. (2018). Proceedings of the IEEE Conference on Computer Vision and Pattern Recognition.

[34] Tan P.S., Lim K.M., Lee C.P., Tan C.H. (2020). 2020 IEEE 2nd International Conference on Artificial Intelligence in Engineering and Technology (IICAIET).

[35] Zhu C., Qin B., Xiao F., Cao Z., Pandey H.M. (2021). A fuzzy preference-based Dempster-Shafer evidence theory for decision fusion. Inf. Sci..

[36] Bian Z.-P., Hou J., Chau L.-P., Magnenat-Thalmann N. (2014). Fall detection based on body part tracking using a depth camera. IEEE J. Biomed. Health Inform..

[37] Hossin M., Sulaiman M.N. (2015). A review on evaluation metrics for data classification evaluations. Int. J. Data Min. Knowl. Manag. Proc..

[38] Poonsri A., Chiracharit W. (2017). 2017 9th International Conference on Information Technology and Electrical Engineering (ICITEE).

[39] Wang B.-H., Yu J., Wang K., Bao X.-Y., Mao K.-M. (2020). Fall detection based on dual-channel feature integration. IEEE Access.

[40] Youssfi Alaoui A., Tabii Y., Oulad Haj Thami R., Daoudi M., Berretti S., Pala P. (2021). Fall detection of elderly people using the manifold of positive semidefinite matrices. J. Imag..

[41] Zheng H., Liu Y. (2022). Lightweight fall detection algorithm based on alphapose optimization model and st-gcn. Math. Probl. Eng..

